# Walking cadence (steps/min) and intensity in 41 to 60-year-old adults: the CADENCE-adults study

**DOI:** 10.1186/s12966-020-01045-z

**Published:** 2020-11-10

**Authors:** Catrine Tudor-Locke, Scott W. Ducharme, Elroy J. Aguiar, John M. Schuna, Tiago V. Barreira, Christopher C. Moore, Colleen J. Chase, Zachary R. Gould, Marcos A. Amalbert-Birriel, Jose Mora-Gonzalez, Stuart R. Chipkin, John Staudenmayer

**Affiliations:** 1 College of Health and Human Services, University of North Carolina at Charlotte, 9201 University City Blvd, Charlotte, NC 28223 USA; 2 Department of Kinesiology, California State University, Long Beach, Long Beach, CA USA; 3 Department of Kinesiology, The University of Alabama, Tuscaloosa, AL USA; 4 School of Biological and Population Health Sciences, Oregon State University, Corvallis, OR USA; 5 Exercise Science Department, Syracuse University, Syracuse, NY USA; 6 Department of Epidemiology, University of North Carolina at Chapel Hill, Chapel Hill, NC USA; 7 Department of Kinesiology, University of Massachusetts Amherst, Amherst, MA USA; 8 Department of Mathematics and Statistics, University of Massachusetts Amherst, Amherst, MA USA

**Keywords:** Physical activity, Pedometer, Accelerometer, Exercise

## Abstract

**Background:**

In younger adults (i.e., those < 40 years of age) a walking cadence of 100 steps/min is a consistently supported threshold indicative of absolutely-defined moderate intensity ambulation (i.e., ≥ 3 metabolic equivalents; METs). Less is known about the cadence-intensity relationship in adults of middle-age.

**Purpose:**

To establish heuristic (i.e., evidence-based, practical, rounded) cadence thresholds for absolutely-defined moderate (3 METs) and vigorous (6 METs) intensity in adults 41 to 60 years of age.

**Methods:**

In this cross-sectional study, 80 healthy adults of middle-age (10 men and 10 women representing each 5-year age-group between 41 to 60 years; body mass index = 26.0 ± 4.0 kg/m^2^) walked on a treadmill for 5-min bouts beginning at 0.5 mph and increasing in 0.5 mph increments. Performance termination criteria included: 1) transitioning to running, 2) reaching 75% of age-predicted maximum heart rate, or 3) reporting a Borg rating of perceived exertion > 13. Cadence was directly observed (i.e., hand tallied). Intensity (i.e., oxygen uptake [VO_2_] mL/kg/min) was assessed with an indirect calorimeter and converted to METs (1 MET = 3.5 mL/kg/min). A combination of segmented regression and Receiver Operating Characteristic (ROC) modeling approaches was used to identify optimal cadence thresholds. Final heuristic thresholds were determined based on an evaluation of classification accuracy (sensitivity, specificity, positive and negative predictive value, overall accuracy).

**Results:**

The regression model identified 101.7 (95% Predictive Interval [PI]: 54.9–110.6) and 132.1 (95% PI: 122.0–142.2) steps/min as optimal cadence thresholds for 3 METs and 6 METs, respectively. Corresponding values based on ROC models were 98.5 (95% Confidence Intervals [CI]: 97.1–104.9) and 117.3 (95% CI: 113.1–126.1) steps/min. Considering both modeling approaches, the selected heuristic thresholds for moderate and vigorous intensity were 100 and 130 steps/min, respectively.

**Conclusions:**

Consistent with our previous report in 21 to 40-year-old adults, cadence thresholds of 100 and 130 steps/min emerged as heuristic values associated with 3 and 6 METs, respectively, in 41 to 60-year-old adults. These values were selected based on their utility for public health messaging and on the trade-offs in classification accuracy parameters from both statistical methods. Findings will need to be confirmed in older adults and in free-living settings.

**Supplementary Information:**

The online version contains supplementary material available at 10.1186/s12966-020-01045-z.

## Introduction

Powered by the rapid pace of consumer-focused commercial advancements in recent decades, wearable technologies capable of detecting movement due to physical activity have made the leap from niche scientific inquiry methods to ubiquitous modes of personal behavior tracking. At the same time, and perhaps due in large part to the commercial surge, step counting (assessing and presenting monitored ambulatory behavior) has steadily gained traction as an intuitively simple approach to communicate physical activity volume (e.g., as steps/day) [1] and more recently, intensity (e.g., as cadence or steps/min) [2]. The 2018 Physical Activity Guidelines Advisory Committee Scientific Report [3] further legitimized the study of step counting and cadence tracking in terms of measuring and modulating ambulatory physical activity. Specifically, this federal document, together with the 2019 Pronouncement from the American College of Sports Medicine [4], call for deliberate new research forays into further quantifying and characterizing the dose-response relationships between step-based metrics and various health outcomes.

Having developed step-based metrics representing volume of daily activity [1], recent scholarly initiatives have turned toward evaluating and deciphering the relationship between cadence and absolutely-defined intensity [2]. Cadence is an essential ambulatory movement pattern and together with stride length delineate speed of ambulation. In this well-known relationship, cadence is the most overtly accessible (and therefore measurable) factor, and especially more so now given the growing availability of wearable technologies capable of tracking this metric in real-time [5]. Absolutely-defined intensity is the rate of energy expenditure required to perform any physical activity and is generally expressed in terms of metabolic equivalent units (METs), standardized to body weight in kg (1 MET = 3.5 mL/kg/min of oxygen consumption) [3, 6]. Based on this definition, an absolute measure of METs is obtained when an individual’s measured VO_2_ in mL/kg/min (mass-specific) is divided by a standardized and presumed resting VO_2_ value of 3.5 mL/kg/min [3, 6]. The relationship between cadence and intensity is strong (*r* = 0.94) [1] and there is evidence of consistent heuristic thresholds indicative of absolutely-defined intensity [7–13]. Research to date, however, has focused on relatively younger adults (i.e., < 40 years of age), with only one study including adults of middle-age (i.e., 41 to 65 years of age) [12]. The study by O’Brien et al. [12] reported that ~ 100 steps/min and ~ 134 steps/min were associated with 3 and 6 METs, respectively, using direct observation to assess cadence and indirect calorimetry to assess intensity. Although their sample represented a broad age range (20–64 years; mean age = 39.4 ± 15.2 years), there were only 43 study participants (58% female). Further, they did not identify cadence thresholds across a broader spectrum of MET-determined levels of intensity (i.e., including 4 and 5 METs). To address these limitations, our previous study in adults 21 to 40 years of age [13] and the present one in 41 to 60 years included a purposefully sex- and age-structured adult sample of young and middle-age (i.e., 10 men and 10 women for each 5-year age group) as well as a determination of cadence thresholds for a more inclusive range of MET values from moderate to vigorous intensity. More research is warranted, as age might modify the cadence-intensity relationship [14], and if so, age-specific cadence thresholds may be necessary to appropriately convey physical activity intensity for public health-related guidelines.

The primary aim of the CADENCE-Adults study was to identify heuristic cadence thresholds associated with increasing intensity during treadmill walking in adults. The first installment from this study further supported 100 steps/min as a consistent heuristic cadence threshold indicative of absolutely-defined moderate intensity (i.e., 3 METs) ambulation in adults 21–40 years of age [13]. That initial research also reinforced the appropriateness of using 130 steps/min as a heuristic cadence threshold indicative of absolutely-defined vigorous intensity ambulation (i.e., 6 METs) in that age group. The purpose of this second installment was 1) to characterize the cadence-intensity relationship in adults of middle-age (i.e., those 41 to 60 years of age), and 2) to identify heuristic cadence thresholds appropriate for this age group.

## Methods

### Overview

CADENCE-Adults is a laboratory-based cross-sectional study of 21 to 85-year-old adults, registered with Clinicaltrials.gov (NCT02650258). The protocol was approved by The University of Massachusetts Amherst Institutional Review Board Data collection and the data collection was carried out in its entirety at the University of Massachusetts Amherst. Recruitment strategies included word-of mouth, locally posted flyers, electronic postings, e-mail blasts, recruitment events, and newspaper and radio advertisements. Telephone screening identified eligibility before scheduling an in-person confirmatory screening process leading up to obtaining written informed consent prior to data collection procedures. The complete methodology, procedures, and inclusion/exclusion criteria have been described elsewhere [13] and are briefly described below. Data collection for this specific age group (i.e., 41 to 60 years of age) took place from January to October*,* 2017.

### Participants

A balanced sex-and-age distribution of 10 men and 10 women for each 5-year age-group between 41 to 60 years (i.e., 41–45, 46–50, 51–55, 56–60 years of age) were recruited, for a total of 80 participants, in order to minimize important sources of bias and improve the generalizability of the findings. The sample size calculation for the CADENCE-Adults study has been described elsewhere [13]. Since the study’s intended focus was on walking cadence, all research participants were independently ambulatory. Exclusion criteria were: Stage 2 hypertension (systolic blood pressure ≥ 160 mmHg or diastolic blood pressure ≥ 100 mmHg), current tobacco use, hospitalization for mental illness within the previous 5 years, body mass index (BMI) < 18.5 kg/m^2^ or > 40 kg/m^2^, cardiovascular disease or stroke, conditions or medications that could affect heart rate response to exercise, pacemakers or other implanted medical devices, and pregnancy. Risk stratification and clinical safety testing procedures [15] have been previously reported [13].

### Measurements and procedures

Participants arrived to the laboratory fasted (at least 4 h) for testing. Information regarding assessment of race/ethnicity (self-reported), standing height, leg length, weight, BMI [16], and waist circumference were described in detail in the initial CADENCE-Adults’ report [13].

Baseline oxygen uptake (VO_2_; mL/kg/min) was measured with the participant sitting on a chair positioned on a stationary treadmill for at least 5 min. Following baseline, participants were instructed to perform up to twelve 5-min treadmill walking bouts at a 0% grade and with incremental changes in speed (from 0.5 mph [13.4 m/min] to a maximum of 6.0 mph [160.9 m/min] in 0.5 mph increments). A table of miles/h, km/h, and m/min conversions is available elsewhere [13]. A period of at least 2-min standing rest was provided between bouts.

Physical activity intensity (i.e., oxygen uptake [VO_2_]) was measured during treadmill testing with a validated [17] portable indirect calorimeter (Jaeger Oxycon Mobile; Vyaire Medical Inc., Chicago, IL). Heart rate was concurrently monitored using a Polar T31 Coded Transmitter chest strap (Polar Kempele, Finland) and the Borg scale [18] was used to capture participant-reported rating of perceived exertion (RPE) during the last minute of each treadmill bout. Steps accumulated in each bout were directly observed and hand-tallied in real-time, with a video recording back-up for step verification purposes. Cadence was then derived as the total steps per bout divided by the bout duration (5 min).

Performance termination criteria of the treadmill test included: 1) transitioning to running; 2) achieving > 75% of age-predicted heart rate maximum [0.75 x (220-age)]; or 3) indicating an RPE > 13. In addition, the participant or the research staff could choose to terminate the protocol based on their own criteria (e.g., for fatigue or safety concerns).

### Data processing and aggregation

Recorded step data were directly read into MATLAB (The MathWorks, Natick, MA) and custom scripts were written to import and process metabolic data in 5-s epochs. To ensure that participants were in a steady state oxygen uptake, mean VO_2_ values for minutes 2:45–3:45 and 3:45–4:45 of each 5-min trial were averaged. Intensity expressed in METs was derived by dividing the mass-specific VO_2_ by 3.5 [19]. Absolutely-defined moderate intensity was interpreted as ≥3.0 METs, and vigorous intensity was ≥6.0 METs [6]. Also, thresholds between moderate and vigorous intensities were defined as ≥4.0 and ≥ 5.0 METs.

### Analytic sample

All 80 enrolled participants provided valid data, resulting in a total of 616 treadmill bouts. Eleven participants ran during their final bout and, consistent with the original report [13], data from these specific running bouts were excluded from the analyses to maintain the focus on cadence-intensity thresholds for walking at 3, 4, 5, and 6 METs. Therefore, the final analytical data set was comprised of 605 treadmill walking bouts. The final analytical data set and corresponding data dictionary can be viewed in Additional files 1 and 2, respectively.

### Statistical analyses

All descriptive and inferential statistical analyses were conducted in R-Studio (version 3.6.2, R Foundation for Statistical Computing, Vienna, Austria). Statistical significance was interpreted as α = 0.05.

Congruent with the analytical processes undertaken in the previous study [13], a segmented regression model with fixed and random coefficients was applied to the data to quantify the cadence-intensity relationship. The model accounts for participant repeated measures, so marginal R^2^ values were reported to describe the model fit, as well as the slopes and 95% confidence intervals (CIs) before and after the breakpoint. To examine any potential influence of leg length, sex, or BMI in the cadence-intensity relationship [9, 20], these variables were included as additional factors in separate segmented regression models. Again, marginal R^2^ values were reported to describe whether models that included leg length, sex, or BMI improved overall prediction.

Also consistent with the previous analyses [13], we used the model’s regression equation to solve for incremental cadence thresholds corresponding to 3, 4, 5 and 6 METs, along with the 95% prediction intervals (PIs). The classification accuracy of each threshold was then evaluated in terms of sensitivity (i.e., the ability of a cadence threshold to accurately identify walking at greater than/equal to the indicated MET value), specificity (i.e., the ability of a cadence threshold to accurately identify walking less than the indicated MET value), positive predictive value (PPV; i.e., the probability that an individual walking at a given cadence threshold would achieve a corresponding predicted intensity level), negative predictive value (NPV: i.e., the probability that an individual walking below a given cadence threshold would not achieve a corresponding predicted intensity level), and overall accuracy (i.e., [true positives plus true negatives] / N) for each regression-identified threshold cadence value. Receiver Operating Characteristic (ROC) curve analyses were also performed. Specifically, four ROC curves were estimated, corresponding to cadence-based classifications of reaching 3, 4, 5 or 6 METs, and an optimal threshold was identified for each curve by selecting the cadence that maximized Youden’s index [21]. Sensitivity, specificity, PPV, NPV, overall accuracy, and area under the curve (AUC) were reported for each ROC analysis. The bootstrap method with 20,000 replicates was used to identify 95% CIs for optimal thresholds and AUCs. AUC values were interpreted as excellent (≥ 0.90), good (0.80–0.89), fair (0.70–0.79), and poor (< 0.70), as previously reported [22].

#### Heuristic cadence threshold determinations

Consistent with our prior installment [13], heuristic cadence thresholds were set using rounded multiples of 5 steps/min informed by the MET-associated optimal thresholds from the segmented model and ROC analysis. Where the two analytical approaches produced discrepant estimates, we considered the trade-offs in terms of sensitivity, specificity, PPV, NPV, and overall accuracy before selecting a single heuristic threshold. We were guided by our previously declared tolerance for error that favors practical applications [13], including recommending, modulating, and/or analyzing ambulatory intensity. To clarify, we selected thresholds while implementing a greater tolerance for false negatives than false positives (both indicators of incorrectly classified bouts). The classification accuracy of heuristic thresholds was subsequently evaluated in terms of sensitivity, specificity, PPV, NPV, and overall accuracy indices relative to the initially identified cadence-intensity estimates.

## Results

### Sample characteristics

Descriptive characteristics are presented in Table 1. The sample was predominantly Caucasian (85%), with a mean ± SD age of 50.2 ± 6.1 years, and a BMI of 26.6 ± 3.7 kg/m^2^. Table 2 presents sample sizes, cadences, VO_2_, and MET values for each treadmill speed. The highest speed reached (by only one participant) was 5.0 mph.

**Table 1:** Descriptive characteristics of the participants

Variable	Men (***n*** = 40)		Women (***n*** = 40)		Total (***N*** = 80)	
	Mean	SD	Mean	SD	Mean	SD
Age (years)	50.2	6.1	50.2	5.9	50.2	5.9
Weight (kg)	83.3	13.3	69.2	11.3	76.3	14.2
Height (cm)	176.9	6.8	165.0	7.1	171.0	9.2
Leg length (cm)	83.8	4.1	77.6	4.4	80.7	5.2
BMI (kg/m^2^)	26.6	3.7	25.5	4.2	26.0	4.0
	n	%	n	%	n	%
BMI classifications						
Normal weight	14	35.0	23	57.5	37	46.3
Overweight	21	52.5	10	25.0	31	38.8
Obese	5	12.5	7	17.5	12	15.0
Race/ethnicity						
White	31	77.5	37	92.5	68	85.0
African-American	1	2.5	1	2.5	2	2.5
Hispanic	1	2.5	1	2.5	2	2.5
Asian	1	2.5	0	0.0	1	1.2
American Indian	0	0.0	0	0.0	0	0.0
Other	2	5.0	1	2.5	3	3.8
Unknown/No response	2	2.5	0	0.0	2	2.5
More than one	2	5.0	0	0.0	2	2.5

BMI categories: normal or healthy weight (18.5–24.9 kg/m^2^), overweight (25.0–29.9 kg/m^2^), obese (≥ 30 kg/m^2^) [16]

**Table 2:** Sample sizes, cadences, VO_2_, and METs for treadmill bouts

Treadmill Speed (mph)	***n***	Cadence (steps/min)	Min-Max	VO_**2**_ (mL/kg/min)	Min-Max	METs	Min-Max
0.5	80	52.5 ± 17.0	31–121	7.0 ± 1.2	4.0–10.9	2.0 ± 0.3	1.1–3.1
1.0	79	70.2 ± 14.2	39–133	8.0 ± 1.2	5.8–12.6	2.3 ± 0.3	1.7–3.6
1.5	79	84.6 ± 10.9	65–141	9.0 ± 1.2	6.4–13.6	2.6 ± 0.3	1.8–3.9
2.0	78	96.1 ± 8.2	77–131	10.0 ± 1.1	7.7–13.1	2.9 ± 0.3	2.2–3.7
2.5	78	105.6 ± 7.0	89–130	11.5 ± 1.1	9.3–14.4	3.3 ± 0.3	2.6–4.1
3.0	78	113.6 ± 6.8	100–131	13.7 ± 1.3	11.0–17.4	3.9 ± 0.4	3.1–5.0
3.5	73	120.1 ± 6.9	106–135	17.0 ± 1.6	13.7–22.1	4.9 ± 0.5	3.9–6.3
4.0	47	128.9 ± 8.8	112–148	21.4 ± 1.7	17.3–26.1	6.1 ± 0.5	5.0–7.4
4.5	12	139.9 ± 9.1	128–152	27.2 ± 2.5	23.7–31.2	7.8 ± 0.7	6.8–8.9
5.0	1	157.4	NA	31.6	NA	9.0	NA

### Segmented regression with random coefficients model

**Fig. 1:**
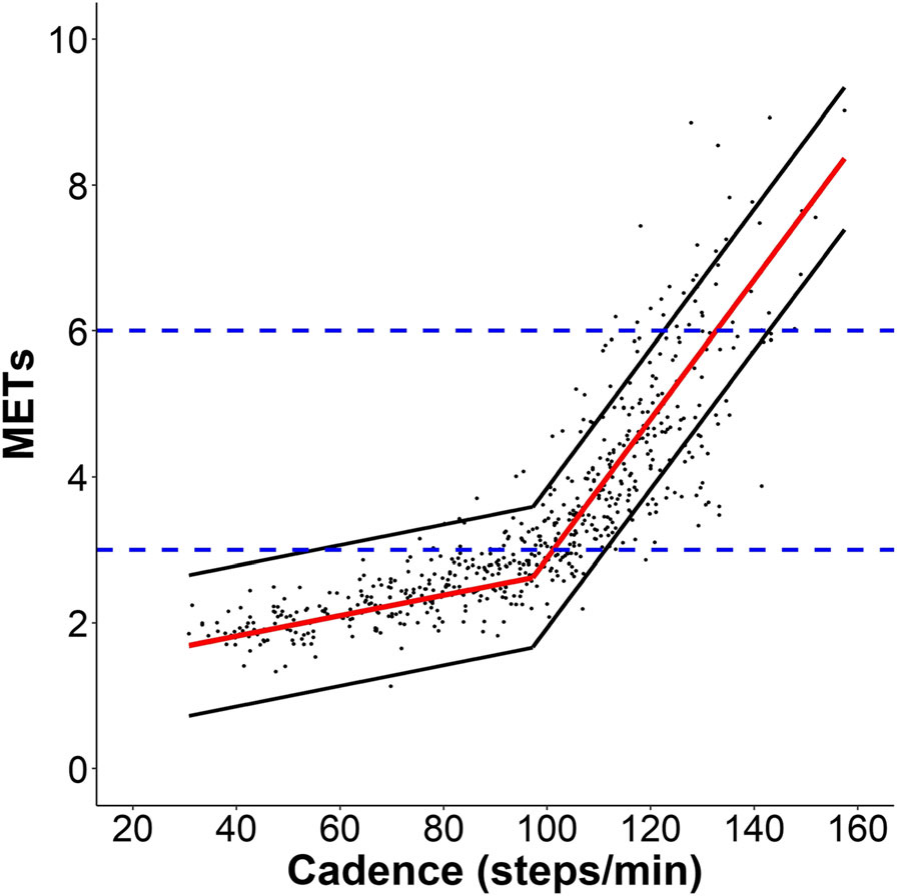
Relationship between cadence and METs using a segmented regression model with random coefficients. Breakpoint is at 97.2 steps/min; marginal R^2^ = 0.81. Red line represents the mean MET values (y-axis) for each corresponding cadence value (x-axis), and the black lines represent the 95% Prediction Intervals. Blue horizontal dotted lines indicate moderate (3 METs) and vigorous (6 METs) intensity, respectively

The cadence-intensity relationship displayed two separate linear regions, with a steeper slope occurring after ~ 100 steps/min. The segmented regression model produced a best fit using a breakpoint at 97.2 steps/min, with a pre-breakpoint slope of 0.014 (95% CI: 0.011, 0.017), post-breakpoint slope of 0.095 (95% CI: 0.092, 0.099), and marginal R^2^ = 0.81 (Fig. 1). Because a segmented regression model was used, we provided two equations: the equation to predict METs from cadences or for intensities below the breakpoint (i.e., ≤ 97 steps/min or ≤ 2.6 METs) was METs = 1.2606 * 0.0141, and that for use with cadences or intensities above the breakpoint (i.e., > 97 steps/min or > 2.6 METs) was METs = − 6.6429 * 0.0954. Adding leg length, sex, or BMI to separate models did not change the breakpoint (i.e., breakpoint at 97.6 for each adjusted model) and neither substantially improved the marginal R^2^ (R^2^ = 0.82 when adding either leg length or sex, and R^2^ = 0.81 when adding BMI). The optimal cadence thresholds (identified using the regression equation) corresponding to 3, 4, 5, and 6 METs were 101.7 (95% PI: 54.9–110.6), 111.8 (95% PI: 101.7–122.0), 122.0 (95% PI: 111.8–132.1), and 132.1 steps/min (95% PI: 122.0–142.2), respectively (Table 3).

**Table 3:** Cadence thresholds (steps/min) for moderate and vigorous intensity based on regression and ROC curve analyses

Intensity (METs)	Measure	Regression thresholds		ROC thresholds		Heuristic thresholds
		Value	95% PI	Value	95% CI	Value
3	Threshold (steps/min)	**101.7**	54.9–110.6	**98.5**	97.1–104.9	**100**
	Se	85.4	–	91.6	–	89.3
	Sp	92.9	–	89.6	–	90.2
	PPV	92.6	–	90.1	–	90.5
	NPV	86.0	–	91.1	–	89.0
	Accuracy	89.1	–	90.6	–	89.8
	AUC	–	–	0.97	0.96–0.98	–
4	Threshold (steps/min)	**111.8**	101.7–122.0	**107.9**	105.5–112.2	**110**
	Se	87.2	–	94.5	–	90.9
	Sp	88.9	–	83.7	–	86.2
	PPV	74.5	–	68.3	–	71.0
	NPV	94.9	–	97.6	–	96.2
	Accuracy	88.4	–	86.6	–	87.4
	AUC	**–**	**–**	0.95	0.93–0.96	–
5	Threshold (steps/min)	**122.0**	111.8–132.1	**115.9**	110.6–117.7	**120**
	Se	70.4	–	90.1	–	79.0
	Sp	92.9	–	85.7	–	90.5
	PPV	60.6	–	49.3	–	56.1
	NPV	95.3	–	98.2	–	96.5
	Accuracy	89.9	–	86.3	–	88.9
	AUC	–	–	0.94	0.92–0.96	–
6	Threshold (steps/min)	**132.1**	122.0–142.2	**117.3**	113.1–126.1	**130**
	Se	52.6	–	97.4	–	55.3
	Sp	97.5	–	82.9	–	96.1
	PPV	58.8	–	27.6	–	48.8
	NPV	96.8	–	99.8	–	97.0
	Accuracy	94.7	–	83.8	–	93.6
	AUC	–	–	0.95	0.93–0.97	–

*AUC* Area under the curve, *CI* Confidence Intervals, *PI* Prediction Intervals, *PPV* Positive Predictive Value, *NPV* Negative Predictive Value, *ROC* Receiver Operating Characteristic, *Se* Sensitivity, *Sp* Specificity

### Receiver operating characteristic analyses

Results of the ROC analysis showing optimal cadence thresholds for incremental levels of intensity are presented in Table 3. Briefly, the optimal cadence threshold for moderate intensity (i.e., 3 METs) was 98.5 steps/min (95% CI: 97.1–104.9) and for vigorous intensity (i.e., 6 METs) was 117.3 steps/min (95% CI: 113.1–126.1). For the intermediate moderate intensities of 4 and 5 METs, optimal cadence thresholds of 107.9 (95% CI: 105.5–112.2) and 115.9 steps/min (95% CI: 110.6–117.7) were observed, respectively. For all intensity thresholds, overall accuracy values were between 84 to 91%, sensitivities and specificities were both > 80%, and the AUCs were excellent (i.e., AUCs ≥0.94).

### Heuristic thresholds

The optimal thresholds derived from both the segmented regression and ROC analyses supported heuristic thresholds (i.e., rounded to the nearest 5 steps/min) of 100 steps/min for 3 METs (102 and 99 steps/min, respectively from regression and ROC analyses), 110 steps/min for 4 METs (112 and 108 steps/min, respectively), and 120 steps/min for 5 METs (122 and 116 steps/min, respectively) (Table 3). Both sensitivity and specificity for heuristic cadence thresholds were > 85% for moderate intensity expressed as 3 and 4 METs and > 79% for 5 METs. Because the optimal thresholds for 6 METs were discrepant across analyses (segmented regression = 117 steps/min, ROC analysis = 132 steps/min), we compared the classification accuracy parameters of 125 and 130 steps/min to inform our ultimate selection of a corresponding heuristic threshold. Using 125 steps/min to classify walking at ≥6 METs resulted in a lower overall accuracy (91%) and PPV (41%) with only a slightly higher NPV (99%) compared to using 130 steps/min (accuracy = 94%, PPV = 49%, NPV = 97%), indicating that 130 steps/min performed comparatively better as heuristic cadence threshold for 6 METs.

**Fig. 2:**
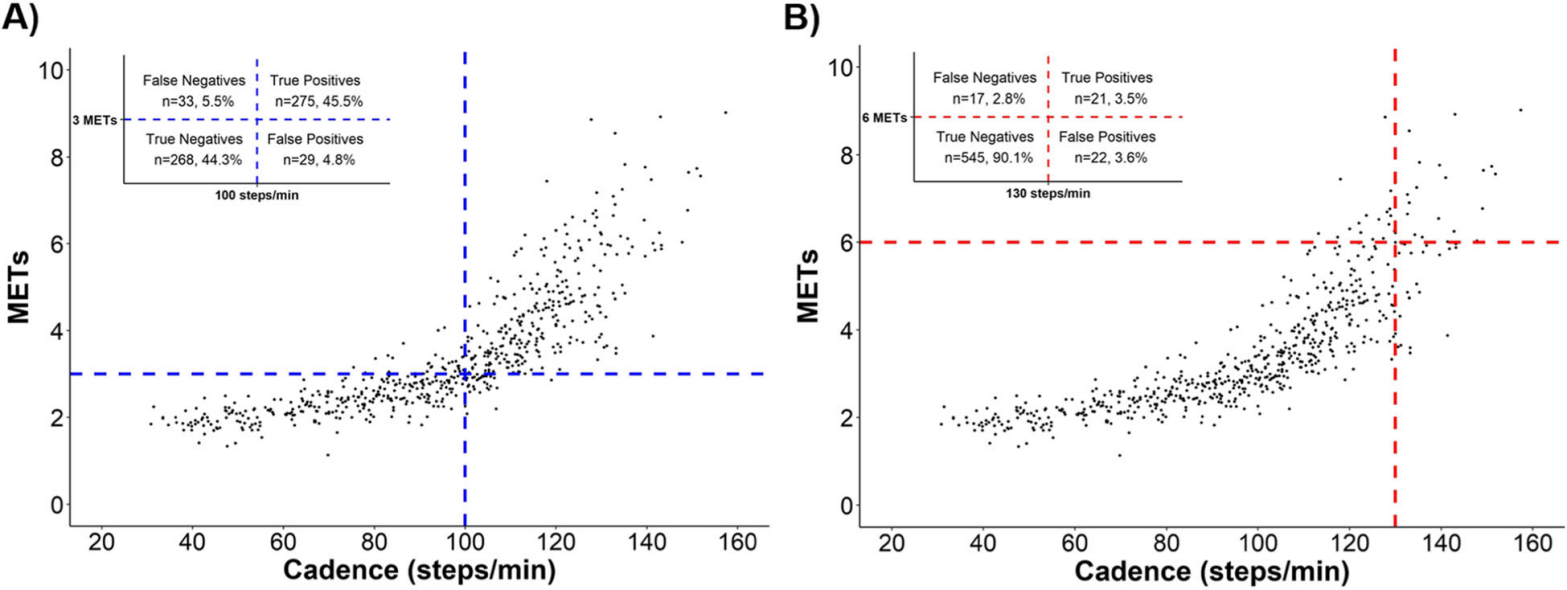
Classification accuracy of heuristic cadence thresholds and MET intensities. A) ≥ 100 steps/min and ≥ 3 METs, B) ≥ 130 steps/min and ≥ 6 METs)

Figure 2 reports the classification accuracy of heuristic cadence thresholds and METs intensities. Ninety percent of all bouts were correctly classified (i.e., overall accuracy: true positives plus true negatives) when the 100 steps/min heuristic cadence threshold was applied for 3 METs and 94% were correctly classified when using the 130 steps/min heuristic cadence threshold for 6 METs. The PPV for achieving a moderate intensity at 100 steps/min was 90.5%, and the PPV for achieving a vigorous intensity at 130 steps/min was 48.8%. Prior to settling on the 130 steps/min heuristic cadence threshold for 6 METs, we also performed a sensitivity analysis to test the classification accuracy of 125 steps/min as an alternative threshold (see Additional file 3). Using 125 steps/min as heuristic threshold, a lower overall accuracy (i.e., 91% bouts correctly classified) was observed in comparison with 130 steps/min. Using 125 steps/min also decreased the PPV by 7.5% (PPV = 41.3%) while NPV was increased by only 1.7% (NPV = 98.7%), indicating that 130 steps/min performed comparatively better as heuristic value.

## Discussion

The primary aim of this study was to establish heuristic cadence thresholds associated with increments of absolutely-defined moderate and vigorous intensity during walking in adults of middle-age. For this purpose, we recruited a balanced sex and age distribution of adults across the targeted age range of 41 to 60 years. Our results establish heuristic cadences thresholds of 100, 110, 120, and 130 steps/minute associated with 3, 4, 5, and 6 METs, respectively, in 41 to 60-year old adults. These heuristic cadence thresholds are intended to: 1) provide researchers with cadence-based goals for use in data analysis and designing walking intervention studies that can elicit expected intensities and associated health benefits; 2) provide clinicians with reliable targets for individuals, and; 3) inform the general public by providing universal cadence-based walking recommendations useful for translating public health guidelines.

The present study in adults of middle-age supports and extends results previously reported based on the same protocol administered to 21 to 40-year-old adults [13] whereby 100 steps/min was also identified as the best heuristic cadence threshold associated with absolutely-defined moderate intensity. This threshold demonstrated an excellent classification of ≥3 METs as evidenced by a probability of achieving absolutely-defined moderate intensity for 90% of those adults of middle-age who walked at a cadence ≥100 steps/min. The optimal cadence thresholds identified using regression and ROC analyses were also very similar to those identified in the previous report focused on younger adults [13]. Also, similar classification accuracy values are observed in both studies (all > 80%), indicating a very high probability that walking at this cadence or above would correspond to achieving an intensity ≥3 METs in adults representing the span between 21 and 60 years of age. To date, most cadence-intensity studies focused on setting such heuristic cadence thresholds have been based on younger adults [7–11, 13], with only a single study by O’Brien et al. [12] including some adults of middle-age in their sample ranging between 20 and 64 years of age. By design, this segment of the CADENCE-Adults study included a balanced sex and age distribution based on 5-year age-grouping (i.e., 41–45, 46–50, 51–55, 56–60 years of age). Together, the present installment (and the previous one in 21 to 40-year-old adults) represents the largest sex and age stratified adult sample to date. Regardless, all of the cadence-intensity studies identified [7–13] reported findings that are consistent with asserting that 100 steps/min is a reasonable and reproducible heuristic cadence threshold associated with absolutely-defined moderate intensity.

To date, a cadence ranging between 125 and 135 steps/min has been associated with absolutely-defined vigorous physical activity (i.e., 6 METs) in younger adult samples (for review, see [2]). The study by O’Brien et al. [12] reported that ~ 134 steps/min was associated with 6 METs [12]. In the present study, the segmented regression and ROC analyses identified 132.1 and 117.3 steps/min, respectively, as optimal cadence thresholds associated with vigorous intensity. Based on these estimates and on previous research in young adults [7, 8, 12], 125 and 130 steps/min were considered as candidates for a heuristic cadence threshold in adults of middle-age. When exploring 125 steps/min, we observed a lower accuracy and lower probability of a bout above this threshold being identified as vigorous intensity in comparison with 130 steps/min. Thus, while 90% of bouts were correctly classified using 125 steps/min as threshold, the use of 130 steps/min correctly classified 94% of bouts. Therefore, for practical reasons and generalizability, we believe that the previously-mentioned factors constitute a rigorous rationale to have ultimately established 130 steps/min as the heuristic cadence threshold for 6 METs. Furthermore, from both a clinical research and a public health point of view, adopting 130 steps/min (vs. 125 steps/min) as a heuristic threshold would ensure identifying more people exclusively exceeding 6 METs and therefore more likely meeting the recommended guidelines for vigorous intensity. This was indicated by 130 steps/min showing a higher specificity than 125 steps/min. Conversely, implementing 125 steps/min as a heuristic threshold for vigorous intensity would allow identification of a greater number of individuals walking at a higher intensity, while increasing the false positive rate (i.e., not necessarily achieving ≥6 METs).

The regression and ROC analyses identified optimal cadence thresholds of 132.1 and 117.3 steps/min for 6 METs, respectively. Again, very similar thresholds were identified in young adults [13] (i.e., 129.1 and 119.5 steps/min). For 6 METs, classification accuracy parameters, although generally consistent across studies, differ more between the regression and ROC analyses performed either in young adults or adults of middle-age. These differences can be considered as normal since both analyses have different assumptions, and therefore different limitations. Thus, regression models may minimize sum of squares for the entire dataset, while Youden’s index from the ROC analysis maximizes sensitivity and specificity for a single threshold. With that said, we believe that a more robust support for the heuristic thresholds (i.e., 100 and 130 steps/min for 3 and 6 METs, respectively) reported in the present study and in the installment in young adults is provided by incorporating both statistical methods.

To the best of our knowledge, this is the first study to also report heuristic cadence thresholds for the intermediary intensity values of 4 and 5 METs in a sample of adults of middle-age. Previous cadence-intensity studies focused on setting heuristic cadence thresholds associated with absolutely-defined moderate and vigorous physical activity intensities (i.e., 3 and 6 METs) only [12]. It is important to note that, while we provide cadence values corresponding with moderate and vigorous intensity thresholds, there is a dose-response relationship between physical activity intensity/volume and health [3]. In fact, the U.S. physical activity guidelines’ recommendation of achieving 150 min per week of moderate intensity or 75 min per week of vigorous intensity physical activity are based on achieving 450 MET-minutes per week [3]. With that said, achieving 4 METs for a given time period will yield greater MET-minutes than 3 METs. Thus, interpreting cadences that correspond with other MET values (e.g., 4 and 5 METs) is important. The first study to propose cadence guidelines for classifying walking intensity speculated that, starting from 100 steps/min, any additional 10 steps/min corresponded to an approximate incremental increase in intensity of 1 MET in young adults [8]. This preliminary finding was supported by the results of the first publication of the CADENCE-Adults study in 21 to 40 year old adults [13] and is further confirmed herein in this sample of 41 to 60 year old adults.

A broad variety of analytical approaches (i.e., linear regressions, multiple regressions, segmented regression, mixed models, ROC) have been used in prior research to investigate the relationship between cadence and intensity [2]. Although previously-reported cadence threshold values were generally similar, apparent discrepancies in point estimates likely reflect differences attributable to variation in sample characteristics and innate properties of the analytical methods themselves. Given such natural variation, heuristic values represent a reasonable reconciliation practice because they are evidence-based (anchored by optimal values from regression and ROC analyses) yet rounded numbers that are intentionally easy to recall. These values can form the basis of general public health recommendations that provide a simple guidance for walking cadence as a strategy to achieve health-related intensity levels. In the present analysis in adults of middle-age, the final heuristic thresholds of 100 and 130 steps/min demonstrated excellent classification (i.e., ≥ 90% accuracy) of absolutely-defined moderate and vigorous intensity ambulation.

Several limitations of this study must be acknowledged. We are aware of the limited precision that any heuristic threshold could have in terms of applicability to any single individual, evident from the inter-individual differences presented herein and in previous studies [9, 11, 23]. However, whereas these previous studies reported that moderate intensity step cadence varies among individuals of different leg lengths and sexes, we found that consideration of these variables did not apparently change the explained variance when accounting for their influence (i.e., 82% of explained variance of intensity when including either leg length or sex to the model compared to 81% of the explained variance by cadence alone). This finding was consistent with our first report focused on young adults [13]. The remaining unexplained variance may be due to anthropometric and physiological factors beyond the scope of this study (e.g., muscle fiber type, etc.). All that being said, it is important to emphasize that the expressed purposes that guided design and analysis was to 1) characterize the cadence-intensity relationship in adults of middle-age (i.e., those 41 to 60 years of age), and 2) identify heuristic cadence thresholds appropriate for this age group. Another limitation to consider is the external validity of thresholds derived from this treadmill-based study when applied to free-living conditions. Notably, we recently reported that a cadence threshold of ≥100 steps/min appears to be a valid heuristic threshold for classifying absolutely-defined moderate intensity during overground walking in young adults [24]. A similar finding is yet to be confirmed in adults of middle-age. Lastly, our results may not be extrapolated to older adults. The final planned installment of the CADENCE-Adults study will confirm or contrast these findings in older individuals 61 to 85 years of age.

## Conclusion

Cadences of 100 steps/min and 130 steps/min are appropriate heuristic cadence thresholds representative of absolutely-defined moderate and vigorous ambulatory intensity (i.e., 3 and 6 METs), respectively, in 41 to 60-year-old adults. In addition, our results support heuristic cadence thresholds of 110 steps/min and 120 steps/min corresponding to 4 and 5 METs, respectively. As such, each 10 steps/min increase is associated with a corresponding 1 MET increase in intensity between 3 and 6 METs, providing a convenient message for public health purposes. Contemporary wearable technologies can now provide instantaneous readings of cadence that would enable individuals to modulate and maintain a cadence ≥100 or 130 steps/min, as desired. However, such devices are not absolutely necessary as individuals could also determine and track their own cadence by simply counting the number of steps accumulated during a 1 min period (or 15 s multiplied by 4, or 10 s multiplied by 6), similar to the manner in which many people are already accustomed to assessing their own heart rate during exercise. Also, if desired, one could match their cadence to a metronome or music as previously shown [25, 26]. Future analyses from the CADENCE-Adults study will also establish heuristic thresholds for walking specifically in older adults of 61 to 85 years of age.

## Supplementary Information

**Additional file 1.** Spreadsheet displaying final analytical data set.

**Additional file 2.** Spreadsheet displaying the data dictionary.

**Additional file 3.** Table displaying a classification accuracy analysis for 125 steps/min as a candidate heuristic cadence threshold for 6 METs.

## Data Availability

All data generated or analyzed during this study are included in this article and its additional files.

## Abbreviations

AUC: Area under the curve
BMI: Body mass index
METs: Metabolic equivalents
mph: Miles per hour
NPV: Negative predictive value
PI: Prediction interval
PPV: Positive predictive value
ROC: Receiver operating characteristic
RPE: Rating of perceived exertion
Se: Sensitivity
Sp: Specificity
VO2: Oxygen consumption
