# Supplementary material for: Walking cadence (steps/min) and intensity in 41 to 60-year-old adults: the CADENCE-adults study
Source: Int J Behav Nutr Phys Act. 2020 Nov 10;17:137. doi: 10.1186/s12966-020-01045-z (PMC7654058; doi:10.1186/s12966-020-01045-z)
Supplement: Supplementary file 3 — Additional file 3. Table displaying a classification accuracy analysis for 125 steps/min as a candidate heuristic cadence threshold for 6 METs. [file 12966_2020_1045_MOESM3_ESM.docx]

**Additional file 3.** Classification accuracy values of 125 steps/min as heuristic cadence threshold for vigorous intensity based on regression and ROC curve analyses.

|  |  | **Regression thresholds** | | | **ROC thresholds** | | | **Heuristic thresholds** | |
| --- | --- | --- | --- | --- | --- | --- | --- | --- | --- |
| **Intensity**  **METs** | Measure | Value | 95% PI^†^ | | Value | | 95% CI | Value | |
|  |  |  |  |  | |  | | |  |
| 6 | Threshold (steps/min) | **132.1** | 122.0 - 142.2 | **117.3** | | 113.1 - 126.0 | | | **125** |
|  | Se | 52.6 | - | 97.4 | | - | | | 81.6 |
|  | Sp | 97.5 | - | 82.9 | | - | | | 92.2 |
|  | PPV | 58.8 | - | 27.6 | | - | | | 41.3 |
|  | NPV | 96.8 | - | 99.8 | | - | | | 98.7 |
|  | AUC | **-** | - | 0.95 | | 0.93 - 0.97 | | | - |
| AUC = Area under the curve, CI = Confidence Intervals, PI = Prediction Intervals, PPV = Positive Predictive Value, NPV = Negative Predictive Value, ROC = Receiver Operating Characteristic, Se = Sensitivity, Sp = Specificity | | | | | | | | | |
